# Independent Component Analysis to Detect Clustered Microcalcification Breast Cancers

**DOI:** 10.1100/2012/540457

**Published:** 2012-04-24

**Authors:** R. Gallardo-Caballero, C. J. García-Orellana, A. García-Manso, H. M. González-Velasco, M. Macías-Macías

**Affiliations:** CAPI Research Group, University of Extremadura, Avenida de la Universidad s/n, 10003 Cáceres, Spain

## Abstract

The presence of clustered microcalcifications is one of the earliest signs in breast cancer detection. Although there exist many studies broaching this problem, most of them are nonreproducible due to the use of proprietary image datasets. We use a known subset of the currently largest publicly available mammography database, the Digital Database for Screening Mammography (DDSM), to develop a computer-aided detection system that outperforms the current reproducible studies on the same mammogram set. This proposal is mainly based on the use of extracted image features obtained by independent component analysis, but we also study the inclusion of the patient's age as a nonimage feature which requires no human expertise. Our system achieves an average of 2.55 false positives per image at a sensitivity of 81.8% and 4.45 at a sensitivity of 91.8% in diagnosing the BCRP_CALC_1 subset of DDSM.

## 1. Introduction

Over a million women worldwide are diagnosed with breast cancer every year, accounting for a tenth of all new cancers and 23% of all female cancer cases. Breast cancer incidence rates vary considerably, with the highest rates in North America and the lowest in Africa and Asia. Around 332,670 new cases of breast cancer occurred in the European Union in 2008 [1] and an estimated 182,460 occur in the USA each year [2]. Although breast cancer is a typically female disease, it may also occur in males, but in a much smaller percentage (1% in the USA, 2010 [2]).

The best way to fight against breast cancer is early-stage diagnosis, for which mammography seems to be the most effective test [3] since lesions can be detected before they are even felt by the patient. Obviously, early-stage detection greatly increases the chances of cure. Unfortunately, even expert radiologists can miss a significant proportion of abnormalities [4]. Moreover, a major fraction of mammographic abnormalities are diagnosed as benign after biopsy [5], obliging these patients to undergo an invasive procedure and to have to wait for the corresponding result. While a second reading of the screening mammograms by a human reader can increase cancer detection rates [6, 7], this has not been proven for second readings of commercial computer-aided detection systems [8].

Many methods have been proposed in the last two decades to achieve a robust mammography-based computer-aided diagnosis (CAD) system. Although there are various types of mammographic abnormality, they can primarily be grouped into either masses or microcalcifications [9]. As noted by Horsch et al. in [10], in both variants, the literature tends to consist of unreproducible studies which provide only a vague description of the mammogram datasets used. Indeed, a premise of the present work is that, currently, the only mammography database that is public and sufficiently large to allow for meaningful and reproducible evaluation of a CAD system is the Digital Database for Screening Mammography (DDSM) [11].

Restricting consideration to papers which provide a reproducible selection of mammograms, one finds the number of papers that address this problem to be really small [10]. One of these works is the study of Kurdziel et al. [12] which uses the 50 images of BCRP_CALC_1 and reports a false positive per image rate (FPi) of 2.8 for a sensitivity value of 0.79. In a second reproducible study on the same dataset, Yuan and Shi [13] report increasing the sensitivity level to 0.9 but the FPi rate rises to a value of 15.4. Better performance values have been reported by nonreproducible studies such as that of Altrichter and Horváth [14](1.6 FPi at a sensitivity of 0.92) or Kang et al. [15] (0.7 FPi at a sensitivity of 0.85).

In the present study, we use an independent component analysis (ICA) feature extractor to diagnose at pixel level the BCRP_CALC_1 mammograms of the USF's Digital Database for Screening Mammography, a reproducible and freely available set of mammograms, to develop a diagnosing system with competitive performance values.

The rest of the paper is organized as follows.  Section 2 describes the main characteristics of the mammogram database and the feature extraction techniques used.  Section 3 presents the main results, and Section 4 the main conclusions.

## 2. Materials and Methods

### 2.1. DDSM and Prototype Selection

The Digital Database for Screening Mammography [11] is a resource openly available to the mammographic image analysis research community. It contains a total of 2620 cases. Each case provides four screening views: mediolateral oblique (MLO) and craniocaudal (CC) projections of the left and right breasts. Therefore, the database has a total of 10,480 images. Mammograms are provided in raw mode and were collected using different scanner models depending on the medical institution where the case was diagnosed. This implies that the mammograms may have different grey level values, sampling rates, and even grey level mapping (linear or logarithmic), so that the first step consists in converting each region of the mammogram to optical density using the calibration equations provided [11].

Cases are categorized into four major groups: *normal, cancer, benign, and benign without callback*. In addition to the regular DDSM volumes, the DDSM website provides the so-called “DoD BCRP Mammography Datasets at USF” comprising four sets [16]. They were created for preliminary evaluation of the performance of CAD algorithms on a common dataset. Two of the four datasets focus on spiculated masses (BCRP_MASS_0 and BCRP_MASS_1) and the other two on clustered microcalcifications (BCRP_CALC_0 and BCRP_CALC_1). Each case included in the BCRP datasets contains at least one malignant lesion.

All cases in the DDSM database were annotated by experienced radiologists providing various BIRADS parameters (density, assessment, and subtlety), a BIRADS abnormality description, and proven pathology. For each abnormality identified, the radiologist draws free-form digital curves defining ground truth regions. Additionally, each case includes other information such as patient age, date of study, and digitization or digitizer's brand.

The present work was carried out using the 100 cases (200 mammograms using the two MLO views of each case) selected in the BCRP_CALC subset. This subset contains uniquely mammograms sampled at 43.5 microns per pixel with two different linear scanners and a spectral resolution of 12 bits. The BCRP_CALC_0 (CALC_0) subset is used to adjust the model, and BCRP_CALC_1 (CALC_1) is used to provide system performance.

Our prototype database was constructed by labeling at pixel level not only microcalcifications but also other typical mammogram structures (eggshell and lucent-centred calcifications, calcified vessels, artifacts, etc.). This step was carried out with the assistance of an experienced radiologist who labeled pixels for the proposed types of structures on all mammograms of the set. We built an initial database of 4638 malignant microcalcification prototypes, 6794 isolated microcalcifications, and more than 100,000 prototypes of completely normal tissue or of some other benign abnormalities.

The first set of prototypes tends to provide erroneous classifications in certain healthy mammogram areas. We thus decided to add more prototypes on those areas with the aim of improving the performance of the pixel classification stage. New prototypes were included as normal pixels using a previously diagnosed mammogram. These new prototypes were included as single-pixel samples in contrast to the previous normal prototypes which were taken by covering small areas of normal tissue. The new normal prototypes seem to provide a broader description of the image space which should be considered as unsuspicious. Hence we decided to discard all previous normal prototypes and preserve only the new 53,334 single-pixel normal prototypes in the following studies.

### 2.2. Independent Components Analysis

Independent component analysis (ICA) [17] is a statistical and computational technique designed to reveal hidden factors that underlie sets of random variables, measurements, or signals. It defines a generative model for the observed multivariate data, typically given as a large sample database. In this model, it is assumed that the data are linear combinations of unknown latent variables, and the system whereby they combine is also unknown. It is also assumed that non-Gaussian latent variables are mutually independent and thus considered independent components of the observed data. These independent components, also called sources or factors, can be found by ICA. ICA-analyzed data may come from very different sources, including digital images, documents, databases, economic indicators, and psychometric measurements. In many cases, the measurements are given as sets of parallel signals or time series in problems known as *blind source separation*.

Thus, suppose that one has *n* signals. The objective is to express the signals registered by the sensors *x*_*i*_ as a linear combination of *n* sources *s*_*j*_, a priori unknown

(1) $$\begin{array}{c} x_{i}=\overset{n}{\underset{j=1}{\sum }}a_{i,j}s_{j}. \end{array}$$

The goal of ICA is to estimate the mixing matrix **A** = (*a*_*ij*_), in addition to the sources *s*_*j*_. This technique can be used for feature extraction since the components of **X** can be regarded as features representing the objects as stated in Chapter 21 of [17].

### 2.3. ICA Feature Extraction and Classification

**Figure 1:**
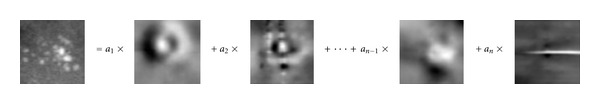
A mammogram region containing microcalcifications expressed as a linear superposition of some ICA-extracted basis functions.

Many basic models in image processing express an image as a linear superposition of some features or basis functions, where the coefficients are different for each image. Some linear transformations widely used in image processing are the Fourier, Haar, and Gabor transforms. Following this model, one can represent a pixel and its neighbourhood in a mammographic image as a series expansion in these functions. The coefficients of this series expansion can then be used as features characterizing the pixel to compose a feature vector which may be used for its diagnosis. One can visualize this concept in Figure 1.

To estimate the ICA basis from images, one needs to collect samples (patches) from the images to model. The collected patches are used to build a data matrix **X** which is the input to the FastICA [18] algorithm. In this algorithm, the data are first centred by subtracting the mean of each column of the data matrix **X**. The data matrix is then *whitened* by projecting the data onto its principal component directions using a prewhitening matrix (**K**). The ICA algorithm then estimates an unmixing matrix **W** so that **X** **K** **W** = **S**, with **S** being the estimated source matrix. Using the previous notation, with $\vec{R}$ being the image associated with a previously centred window, one can obtain the feature vector ($\vec{F}$) that characterizes the window as $\vec{F}=KW\vec{R}$.

The number of features to obtain and the size of the sampling window are selected a priori. Therefore the optimum ICA configuration for the problem must be determined using a supervised procedure. Each feature extractor requires a set of ICA matrices to be generated. We created them using samples centred on the previously defined prototypes. Thus, working at pixel level, we compute a wide range of ICA features (from 2 to 80) for different window sizes (from 3 to 81 pixels wide) over a set of more than 65,000 testing samples. The ICA matrix generation was carried out using the FastICA implementation available for the R statistical environment [19].

Once the ICA basis has been obtained, one can obtain a feature vector that characterizes the pixel under study and its neighbourhood by selecting the window size and the number of features to extract. We used an artificial neural network (ANN) to classify the feature vectors obtained, in particular, a classical feed-forward multilayer perceptron with a single hidden layer trained with a variation of the back-propagation algorithm named Resilient Back-Propagation (RPROP) [20] to obtain faster convergence. The number of neurons in the hidden layer is allowed to grow from 50 to 200 in order to improve the success rate of the classifier.

In this step, the objective is to optimize microcalcification detection in mammograms. Hence, for each combination of ICA window size and feature number, one needs to compute the success rate in microcalcification detection of each classifier. Due to the heavy demand on computational resources required to select the best ICA feature extractor and classifier, all subsequent batch calculations were carried out on a 48 quad-core distributed PC cluster.

### 2.4. Generation of Regions of Interest

To determine the location of a cancer defined by a microcalcification cluster in a mammogram, one needs to generate regions of interest (ROIs) in those areas where the number of microcalcifications is greater than 3 in an one-square-centimetre neighbourhood [21]. Therefore, once the microcalcification detection phase has been optimized, each mammogram is processed to obtain an image with the location of microcalcification candidates. This image is filtered to discard spurious microcalcifications (isolated pixels or artifacts). The resulting image is processed by an ROI builder system which groups neighbouring microcalcifications into disjoint zones of interest.

**Figure 2:**
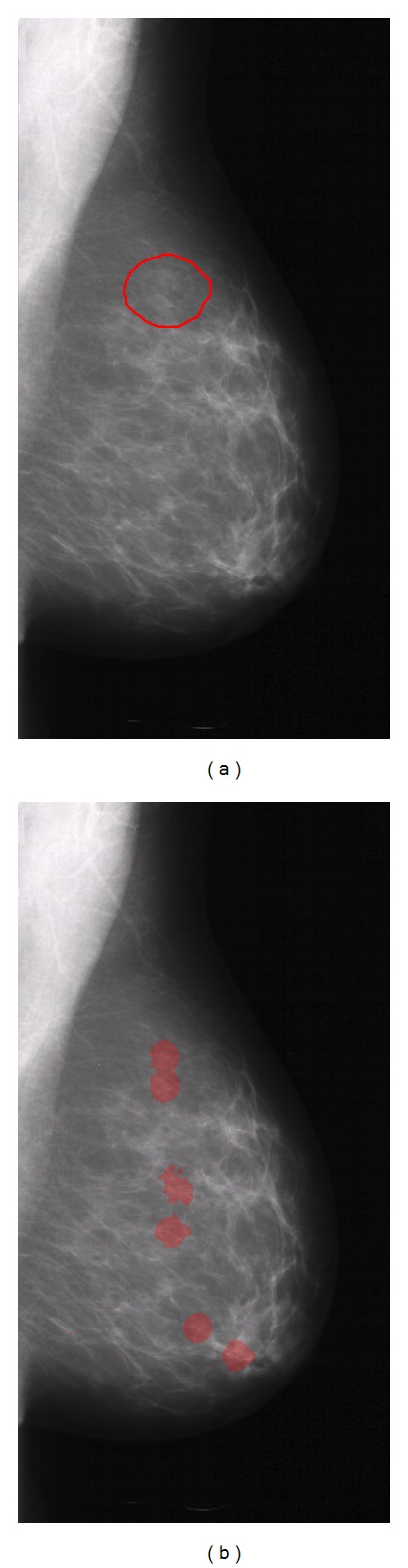
(a) Original DDSM mammogram with the expert radiologist's mark surrounding the microcalcification cluster. (b) Mammogram showing the regions of interest generated by our system. The upper ROI accounts for the true abnormality.

The first step of the ROI builder consists in an object detector that isolates microcalcifications and computes their sizes. The second step consists of grouping the objects by means of a density map. For each pixel of the mammogram, the number of microcalcifications in the previously defined neighbourhood area is computed. After that, the density map is filtered, retaining only those zones which have a microcalcification density value equal to or greater than 3.  Figure 2(b) provides a sample of the regions of interest obtained with this procedure on a mammogram.

### 2.5. Computing Performance

The performance of mammographic CAD systems is evaluated by means of such parameters as sensitivity, specificity, and false positives per image (FPi). Sensitivity (2) or the true positive rate measures the proportion of actual positives which are correctly identified as such. And FPi measures the mean value of false positives (FP) that are generated for a test set (see (3))

(2) $$\begin{array}{c} \text{sensitivity}=\frac{\text{TN}\#}{\text{TN}\#+\text{FN}\#}, \end{array}$$

(3) $$\begin{array}{c} \text{FPi}=\frac{\text{FP}\#}{\text{image}\#}. \end{array}$$

The criteria chosen in our implementation to define the possible outcomes of our system are as follows. Each generated possible ROI in a mammogram is tagged as true positive (TP) if it overlaps with the annotated region provided in the DDSM database (if present). Analogously the ROI is tagged as false positive (FP) if there exists no overlap with an actual malignancy of the mammogram or the mammogram is healthy. On the contrary, if DDSM contains malignant ROIs for a mammogram and our system fails to find them, we compute all those ROIs as false negatives (FN). And finally, a successfully diagnosed healthy mammogram is considered a true negative (TN). 

A graphical method known as free-response operating characteristic (FROC) is also used to evaluate the performance of CAD system. A computer-based detection algorithm attempting to locate a target in a digital image may provide more than one suspicious zone in an image, in most cases only one being the correct target while the others are false positives. FROC [22] is a variant of receiver operating characteristic analysis [23] needed to be able to take into account the FP data. For an FROC curve, the hit rate is expressed as a proportion, but unlike ROC curves the FP rate is expressed as the mean number per image. FROC curves provide a clearer picture of how a system is operating than a conventional ROC curve. However, no very useful measure of sensitivity can be derived from them.

## 3. Results and Discussion

Any combination of ICA window size and number of features to extract generates an input feature extraction configuration to be evaluated. To train the neural classifier, we defined a dataset of pixel locations for which ICA-extracted feature vectors should be obtained for each input configuration. The dataset was split into three disjoint subsets: learning, validation, and test according to pixel origin (CALC_0 or CALC_1). The validation subset is to avoid overtraining and improves the network generalization capabilities [24].

The Stuttgart neural network simulator (SNNS) [25] environment was used to generate and train the network. The high number of input configurations to optimize led us to use SNNS's kernel function facilities to implement the network training procedures in standalone executables that could be run in a distributed Beowulf cluster [26]. 

To achieve an optimal network configuration, we made a sweep of the number of neurons in the hidden layer. We built and trained several network configurations with a number of hidden neurons ranging from 50 to 200 in steps of 50. Furthermore, each configuration was repeated four times, performing a random initialization of the neuron weights in each repetition, and retaining the classifier that provides the greatest success rate. Finally, the test subset, which contains input vectors extracted from CALC_1 prototypes, was used to provide performance results of the microcalcification detection subsystem. 

The main objective of the study was to obtain a system that relies mainly on image processing features, but we also studied input configurations that included the patient's age as an additional feature. The age of the patient was scaled linearly from the range [27,91] to the range [−1, +1] for inclusion in the feature vector.

### 3.1. Detection Performance on Complete Mammograms

We trained 7011 neural classifiers with different input configurations and prepared 85 experiments to evaluate the performance on the CALC_1 subset. Each experiment provides different performance values depending on the network-analyzing function selected, so that the 85 experiments required repeating the diagnosis procedure 1705 times. These numbers may give an idea of the immense number of data to be analyzed.

Table 1 lists the five best results in terms of lowest FPi per image at a sensitivity level higher than 80% on the 50 cases of the CALC_1 subset (100 MLO mammograms). As can be seen, the lowest FPi value obtained in this case is 2.55, slightly less than that reported by Kurdziel et al. [12] (2.8). It was noteworthy that the ICA window sampling size required for the best system was really small (565.5 *μ*m on a side) and that the feature vector consisted of only 40 features. This seems to suggest that microcalcification cluster detection is a highly local task. The patient's age appears in the third configuration, but its presence was not required to reach the highest success rates, thus allowing a system to be used that is completely based on image-extracted parameters.

The analyzer column in Tables 1 and 2 specifies the analyzing function used to classify the output of the neural network. Here, 402040 stands for the “402040” network activation rule [25] with parameters *l* = 0.45 and *h* = 0.55, and WTA stands for winner takes all, meaning that the classification depends on the neural network unit with the highest output and the value of the parameter *h*. The value of the parameter *h* is zero for WTA0, 0.5 for WTA1, 0.55 for WTA2, and 0.6 for WTA3.

Increasing the sensitivity value requirement to 90%, one observes a rise in the mean number of false positives per image generated. The lowest FPi value obtained by a system was 4.45 at a sensitivity of 91.8% (see Table 2), a significantly lower value than the 15.4 reported by Yuan and Shi [13]. It is noteworthy that the age of the patient seems to play a major role when high sensitivity values are required. Nevertheless, again both the size of the ICA sampling window and the number of ICA features to use remain at low values, reducing the computational demand.

**Figure 3:**
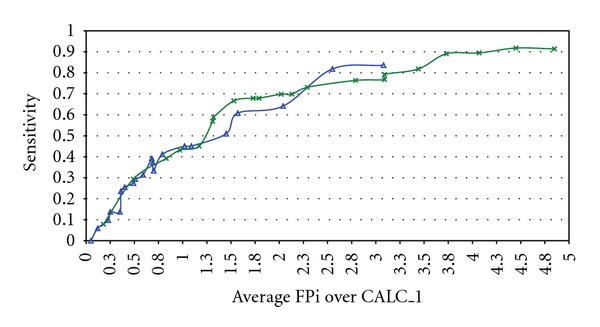
Free-response operating characteristic curves for systems with the feature extraction configurations ICA-D13C40 (blue trace) and ICA-D17C20+AGE (green trace).

Figure 3 shows the FROC curves for the systems that provide the lowest FPi values at 80% and 90% sensitivity levels and working on the CALC_1 subset with the 21 predefined analyzing functions. The blue trace shows the FROC performance of the system with the lowest FPi at a sensitivity level higher than 80%, and the green trace shows the same but for a sensitivity level higher than 90%.

## 4. Conclusions

Although the computer-aided detection of breast cancer is a fairly well-established field of research, the complexity of mammographic images has led to there being really few reproducible studies of diagnosis based on microcalcification cluster detection. Independent Component Analysis seems to constitute a very suitable approach to the efficient detection of clustered microcalcifications, improving published reproducible performance results by a factor of from 8.9% (at a sensitivity higher than 79%) to more than 71% (for sensitivities higher than 90%).

The relative small area required for the ICA windows that provide the best microcalcification cluster detection seems to suggest that this task is accomplished by ICA analysis at a relatively small scale, neglecting information from pixels located at middle or long distances.

The use of the patient's age as a parameter seems to be significant for high sensitivity requirements, but of negligible effect at lower sensitivity levels. In either case, its use would appear to be acceptable because it is easily obtainable, whereas other proposals sometimes use expert-provided DDSM parameters such as breast density or even the assessment of the abnormality to provide a diagnosis.

## Figures and Tables

**Table 1:** Performance results on the BCRP_CALC_1 subset of the five system configurations that generate the lowest false positives per image and provide a sensitivity higher than 80%.

Configuration	Analyzer	FPi	Sensitivity (%)
ICA-D013C040	WTA1	2.55	81.8
ICA-D013C040	WTA0	3.08	83.6
ICA-D015C020+AGE	402040	3.25	81.4
ICA-D015C024	WTA1	3.40	84.2
ICA-D017C020+AGE	WTA3	3.44	81.8

**Table 2:** Performance results on the BCRP_CALC_1 subset of the five system configurations that generate the lowest false positives per image and provide a sensitivity higher than 90%.

Configuration	Analyzer	FPi	Sensitivity (%)
ICA-D017C020+AGE	WTA1	4.45	91.8
ICA-D017C020+AGE	WTA0	4.85	91.3
ICA-D019C020+AGE	WTA1	4.97	91.5
ICA-D015C010+AGE	WTA3	5.40	91.9
ICA-D015C010+AGE	WTA2	5.58	92.0
